# Intraspecific variation in the duration of epigenetic inheritance

**DOI:** 10.1101/2025.06.04.657799

**Published:** 2025-06-07

**Authors:** François Frejacques, Marie Saglio, Mohammed Aljohani, Christian Froekjaer-Jensen, Lise Frézal, Marie-Anne Félix

**Affiliations:** 1: IBENS, Department of Biology, Ecole Normale Supérieure, CNRS, Inserm, PSL Research University, Paris, France; 2: Bioscience Program, Biological and Environmental Science and Engineering (BESE), King Abdullah University of Science and Technology (KAUST), Thuwal, 23955-6900, Kingdom of Saudi Arabia; 3: Present address: Department of Developmental and Stem Cell Biology, Institut Pasteur, Paris, France; 4: Present address: Institut Pasteur, Université Paris Cité, Unité des Bactéries pathogènes entériques, Paris, France; 5: Present address: Department of Genome Sciences, University of Washington, Seattle, WA 98195, USA; 6: First authors, alphabetical order

**Keywords:** epigenetic inheritance, evolution, *C. elegans*, small RNAs

## Abstract

Epigenetic inheritance is generally less stable across generations than DNA sequence-based heredity. One common form of epigenetic inheritance, found in plants, fungi, and animals, is the transgenerational memory of gene silencing mediated by small RNAs. These small RNAs can be amplified through RNA-dependent RNA polymerases, thus maintaining the silenced state across multiple generations. Such molecular mechanisms raise questions regarding their natural variation and evolutionary impact. We here ask whether the presence and duration of epigenetic inheritance display genetic variation within a species. We use the ability of the nematode *Caenorhabditis elegans* to silence genes for multiple generations after an initial RNA interference trigger. We find that the presence and the duration of silencing in number of generations differ across *C. elegans* wild strains. Strikingly, several wild strains show no memory, while some display a longer memory than the reference strain. Natural DNA sequence polymorphisms, such as in the *set-24* and *drh-1* genes, affect epigenetic memory of an external trigger, demonstrating that intraspecific DNA sequence evolution affects the duration of epigenetic inheritance. We further show that the duration of silencing memory in wild strains is quite robust to environmental variation such as diet and passage through larval diapauses, but not to temperature variation. Altogether, these results demonstrate intraspecific diversity in the regulation of small RNA-based heredity, a prerequisite for selection acting on genetic variants affecting epigenetic inheritance duration.

## Introduction

Various molecular mechanisms of epigenetic inheritance have been uncovered over the last decade across biological kingdoms, thus renewing the interest about the importance of such non-conventional inheritance in evolution (Lind and Spagopoulou, 2018; Bonduriansky and Day, 2018; Adrian-Kalchhauser et al., 2020; Duempelmann, Skribbe et al., 2020; Ashe et al., 2021). Two features distinguish epigenetic variation from DNA sequence variation: 1) epigenetic variants may originate not only stochastically but also through environmental induction; 2) epigenetic variants are generally less stable over generations than DNA sequence. We here focus on this latter point, regarding the stability of epigenetic variants across generations. At a large macroevolutionary scale, this duration ranges from long-term stability of methylated epialleles in plants (Cubas et al., 1999; Weigel and Colot, 2012; Miska and Ferguson-Smith, 2016; Fitz-James and Cavalli, 2022) or of paramutation in *Drosophila* (de Vanssay et al., 2012) to a lack of clear demonstration of a multigenerational memory in mammals beyond parental or grandparental effects (Heard and Martienssen, 2014). In the midst of this stability spectrum lies small RNA silencing in the nematode *Caenorhabditis elegans*, with a few generations of memory of the initial trigger in a commonly used experimental paradigm. This intermediate range of a few generations offers a particularly favorable case to study its variation within the species and the possible evolutionary significance of this variation.

Specifically, the *C. elegans* reference strain N2 is able to transmit the memory of silencing by double-stranded RNA, for example after an initial trigger of exogenous RNA interference (RNAi). The primary small RNAs are amplified in secondary small RNAs by RNA-dependent RNA polymerases (Sijen, Fleenor, Simmer et al., 2001; and Fire, 2007; Vasale, W. Gu et al., 2010; Pak et al., 2012; Tsai et al., 2015; X. Chen, K. Wang, Mufti et al., 2024). This amplification in principle can maintain the memory of the initial signal over several generations even after it has been removed (Gu et al., 2012; Billi et al., 2014; F. Xu, X. Feng et al., 2018; Shukla et al., 2021; Ouyang et al., 2022). Many factors required for the inheritance of small RNA pools were discovered using laboratory genetic screens in the N2 reference background (e.g. Grishok et al., 2000; Vastenhouw et al., 2006; Buckley, Burkhart et al., 2012; Lev, Seroussi et al. 2017; Spracklin et al., 2017; Du, Shi et al., 2023; Chen and Phillips, 2024). The RNAi memory mechanism is distinct from the RNAi mechanism itself (Grishok et al., 2000; Buckley, Burkhart et al., 2012).

This molecular mechanism of amplification and inheritance along generations raises questions regarding its ecological and evolutionary context. A prerequisite for studying how natural selection may act on epigenetic inheritance duration is to detect intraspecific diversity in the initiation and duration of epigenetic inheritance. The aim of the present work is to measure intraspecies variation for RNAi memory duration in *C. elegans*. Note, that we will not explore the natural variation in epialleles (here small RNA sequence representation), nor which external ecological factors may trigger initial variation in small RNA pools (Baugh and Day, 2020), but how long the memory of such triggers remains.

We probe natural strains of *C. elegans* for their transmission of an initial RNAi trigger and find that they differ in both occurrence and duration of their RNAi memory. We show that natural DNA sequence polymorphisms in the *set-24, drh-1* and *eri-6/7* genes may affect epigenetic memory of silencing. In addition to genetic variation, we find that the RNAi memory duration varies with temperature and is quite insensitive to our other tested environments, including starvation, passage through dauer diapause or bacterial diet. More importantly, the detection of intraspecific genetic variation for the presence and duration of RNAi memory means that, like any quantitative trait, epigenetic inheritance can respond to standard natural selection.

## Results

### The duration of RNAi silencing memory varies among *C. elegans* wild isolates

**Figure 1:**
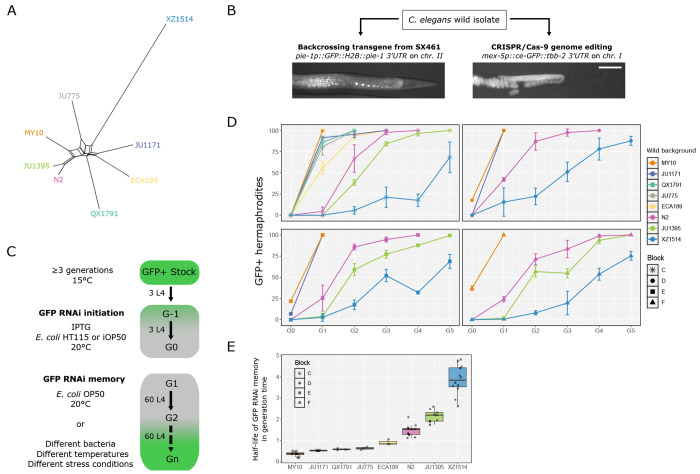
Variation in the duration of RNAi memory among *C. elegans* natural isolates. **(A)** Haplotype network of the *C. elegans* natural isolates used in this work. The network is based on genome-wide single-nucleotide polymorphisms from CaeNDR (Crombie et al., 2019) and built using SplitsTree. **(B)** Introduction of germline-expressed transgenes into C. *elegans* natural isolates, either through introgression by successive backcrosses or introduction at the same genomic site by CRISPR/Cas9-mediated editing. The two transgenes differ in *cis*-regulatory sequences, coding sequences and 3’UTR. The *pie-1p* transgene drives expression of GFP fused to histone H2B. The *mex-5p* transgene drives expression of a *ce-GFP* sequence with two NLS sequences and is devoid of predicted piRNA sites. The fluorescence micrographs show their different patterns of GFP expression. Bar: 100 μm for both pictures. **(C)** Schematic of the GFP silencing memory assay. RNAi is triggered by feeding a stock of animals expressing GFP (as indicated by the green color) with *E. coli* bacteria expressing double-stranded RNA against the corresponding GFP sequence for two generations (called G-1 and G0). The two transgenes strongly differ in coding sequences, and thus distinct RNAi clones were used to silence each transgene. Experimental variations during RNAi initiation or the memory assay are indicated. **(D)** Four independent experiments assaying RNAi memory variation in different isolates with the *mex-5p::ce-GFP::tbb-2* transgene inserted on chromosome I. We call each of these four independent experiments a block, lettered here C, D, E and F. Three replicates were run for each line for each block (see Table S2 for raw results). In these experiments, RNAi was initiated with *E. coli* HT115 bacteria transformed with pMNK25. For the sake of simplicity, we indicate the name of the wild isolate background rather than that of the genome-edited strain (see Table S1 for the corresponding strain name). The rank order of the strains is highly reproducible. On the graph, the lines follow the means of the three replicates and error bars represent their standard deviation (SD). **(E)** Boxplot showing the estimated half-lives of *gfp* silencing memories extracted from the data in (D) (see Table S3 for raw values). Generalized linear mixed-model (glmm) statistics based on these values (excluding strains tested once) demonstrated a strong effect of the strain on the dynamics of *gfp* desilencing over generations: *p* < 2x10^−16^.

We first selected genetically divergent *C. elegans* wild isolates that are sensitive to RNAi. Based on the data by (Paaby et al., 2015; Crombie et al., 2019) and our prior work with the MY10 strain (Frézal et al., 2018), we focused on the eight wild strains shown in Fig. 1A. We used two distinct transgenes to assay small RNA silencing and two methods to introduce them into wild genetic backgrounds. First, we introduced a single-copy *pie-1p::GFP::H2B::pie-1* transgene by repeated backcrosses to the target wild isolate background (Ashe, Sapetschnig et al., 2012). With this transgene, fluorescence is restricted to the germline nuclei and strictly nuclear because of the histone protein fusion (Fig. 1B). Second, we used CRISPR/Cas9 genome editing to introduce at the same site in all wild isolate backgrounds a specifically engineered *mex-5p::ce-GFP::tbb-2* transgene, optimized for high expression and devoid of piRNA sites (Aljohani et al., 2020). As a result, the germline-specific localization of this *C. elegans* enhanced GFP (*ce-gfp*) is brighter, and fluorescence also present in the cytoplasm because, despite the nuclear localization signals, the protein is less retained in the nucleus than with the histone fusion (Fig. 1B).

Using the protocol from (Ashe, Sapetschnig et al., 2012; Lev, Seroussi et al., 2017) (Fig. 1C), we assayed the duration of RNAi memory in the reference N2 background in parallel with the *pie-1p::GFP* and *mex-5p::ce-GFP* transgenes. After RNAi initiation, both transgenes were initially silenced in all animals (Fig. S1A). Once the RNAi trigger was removed, full silencing was maintained in the progeny (generation G1). Starting at the third generation, a fraction of the population re-expressed GFP and this percentage increased through generations. The two transgenes showed similar dynamics; however as can be expected from the lack of piRNA sites, the RNAi memory was maintained for a shorter time with the *mex-5p::ce-GFP* transgene (Fig. S1C). We further mainly used the *mex-5p::ce-GFP* transgene but report below on the convergent results using both methods.

Our first aim was to test whether wild genetic backgrounds differed in their RNAi memory duration. After a two-generation exposure to *gfp* RNAi from *E. coli* HT115 bacteria, we found that the *mex-5p::ce-GFP* transgene was fully silenced in all isolates, with the exception of some individuals of the MY10 strain, and that the duration of RNAi memory greatly varied among wild genetic backgrounds (Fig. 1D–E, Table S2, Table S3). Once the RNAi trigger was removed, a fraction of the population in the N2 reference re-expressed GFP at the first generation and this percentage increased until fluorescence was fully recovered in all individuals at the third or fourth generation. Some wild isolates, such as MY10, JU1171 or QX1791, did not, or hardly, transmit parental GFP silencing to the first generation. Other isolates, such as JU1395 or XZ1514, displayed an increased duration of RNAi memory compared to N2. The results were consistent across three replicates per experiment and four distinct experiments, which we call “blocks”, lettered C, D, E and F (Fig. 1D, Table S2). We plotted in Fig. 1E estimated half-lives of GFP silencing memory. Using this estimate, we found significant differences for GFP desilencing dynamics across wild genetic backgrounds (generalized linear mixed model or glmm, *p* < 2x10^−16^).

We further asked whether the variation among isolates could be reproduced using the *pie-1p::GFP* transgene. As expected, given the possible silencing by piRNAs of the *pie-1p::GFP::H2B::pie-1* transgene, all displayed increased RNAi memory with this transgene compared to its *mex-5p::ce-GFP::tbb-2* counterpart (Fig. S1D–E). Most importantly, the relative rank of RNAi memory duration of the tested wild isolates was well conserved (Fig. S1D), showing that they differ in the duration of their RNAi silencing memory.

### The *set-24* DNA sequence polymorphism affects RNAi memory

**Figure 2:**
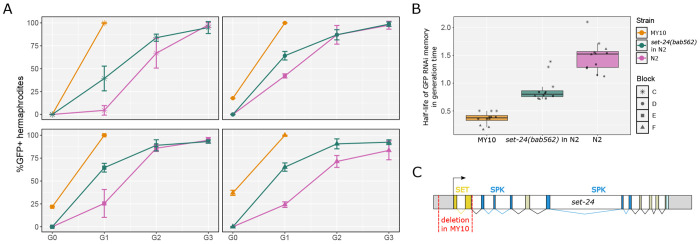
A natural DNA sequence polymorphism in the *set-24* gene affects RNAi memory. **(A)** Effect on RNAi memory of the *set-24(bab562)* allele using the *mex-5::ce-GFP* transgene in the N2 genetic background. The four panels correspond to the same four blocks as in Fig. 2A. Lines of each graph follow the mean of three replicates and the bars represent the standard deviation of the replicates. **(B)** Boxplot showing the estimated half-lives of GFP RNAi memory. Glmm analysis based on these values followed by a Tukey pairwise comparison indicates a statistical difference for the median RNAi silencing memory between N2 and the strain carrying the *set-24(bab562)* allele in N2 background: *p* < 1x10^−4^, as well as between the latter and the MY10 strain: *p* < 1x 10^−4^. **(C)** Schematic depicting the *set-24* gene and the location of the natural deletion in the MY10 genetic background.

We then aimed to determine whether DNA sequence polymorphisms could explain variation in RNAi memory, thus ruling out other types of hereditary variation that may influence this phenotype. For this, we leveraged our prior knowledge of natural variation in another multigenerational phenotype. The mortal germline phenotype (Mrt) is a multigenerational sterility process whereby lines become sterile after several generations (Ahmed and Hodgkin, 2000). Several mutations affecting small RNA pathways, chromatin remodeling and RNAi memory cause a temperature-sensitive sterility phenotype in the N2 background (Ahmed and Hodgkin, 2000; Buckley, Burkhart et al., 2012; Lev, Seroussi et al., 2017; Spracklin et al., 2017). Interestingly, some wild isolates cultured in laboratory conditions also display multigenerational sterility, which is counteracted by association with natural bacteria (Frézal et al., 2018; Frézal, Saglio et al. 2023). An example is the MY10 strain, which becomes sterile after only two to three generations at 25°C. (Frézal et al., 2018) showed that a partial deletion of the *set-24* gene in MY10 (Fig. 2C) was a major effect variant causing a Mrt phenotype. *set-24* encodes a SET-domain protein likely binding modified histones. Given the relationship between RNAi memory and the Mrt phenotype, and the lack of RNAi memory of the MY10 background (Fig. 1D–E), we asked whether this *set-24* polymorphism (Fig. 2C) affected RNAi memory.

For this, we engineered in the N2 background the *set-24(bab562)* allele that mimics the natural deletion from the MY10 isolate (called *set-24*(*mfP23*)) and crossed it to the *mex-5p::ce-GFP::tbb-2* transgene. We found that the *set-24(bab562)* allele significantly reduced RNAi memory compared to the N2 genetic background (Fig. 2A–B). This *set-24* polymorphism explained only part of the difference in RNAi memory between the N2 and MY10 backgrounds, suggesting that further polymorphisms may underlie some RNAi memory difference between the two strains. Altogether, this result demonstrates that DNA sequence evolution affects epigenetic memory.

### The intermediate frequency *drh-1* polymorphism affects RNAi memory

Given that the derived *set-24* deletion allele is rare in the wild (Frézal et al., 2018), we wondered whether more common and thus potentially more evolutionary stable polymorphisms could underlie natural variation in the duration of RNAi memory. *C. elegans* antiviral defense mechanisms rely in part on recognition of viral double-stranded RNA (dsRNA) and production of small RNAs targeting the virus (Ashe, Bélicard, Le Pen, Sarkies et al., 2013; Coffman et al., 2017; Sowa et al., 2020; Batachari et al., 2024). As the machinery required to recognize and initiate antiviral response is in part the same as that of RNAi (Tabara et al., 2002; Consalvo et al., 2024), we wondered whether polymorphisms found in the double-stranded RNA antiviral pathway could also affect RNAi memory.

**Figure 3:**
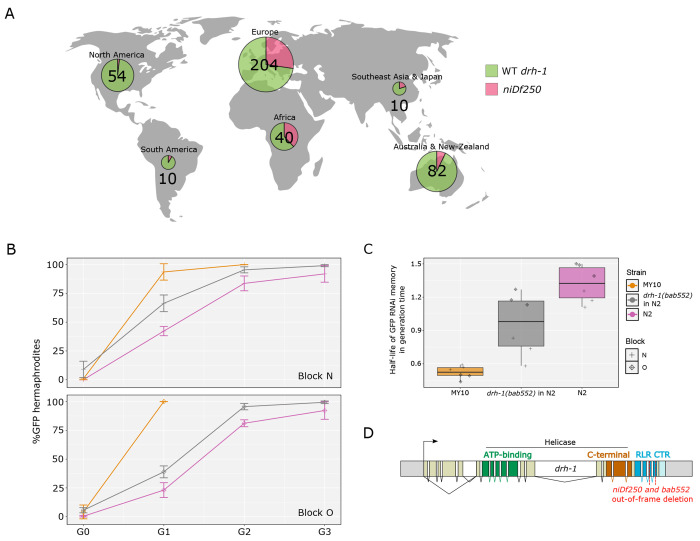
A common natural DNA sequence polymorphism in the *drh-1* gene affects RNAi sensitivity and memory. **(A)** Diagram depicting the proportion of isotypes possessing the 159 bp deletion in *drh-1* by world region. Numbers shown correspond to the number of isotypes sequenced by continent from CaeNDR. As no isolate sampled in Hawaii presents this polymorphism, Hawaii was excluded from the representation. Created with BioRender.com. **(B)** Effect on RNAi memory of the *drh-1(bab552)* allele using the *mex-5::ce-GFP* transgene in the N2 genetic background. Panels correspond to blocks N and O. In these two blocks, *E. coli* iOP50 bacteria transformed with pFF1 (see Methods) were used to initiate RNAi against *gfp*. Lines of each graph follow the mean of three replicates and the bars represent the standard deviation of the replicates. **(C)** Boxplot showing the estimated half-lives of GFP RNAi memory of the strains tested in blocks N and O. Glmm analysis based on these values followed by a Tukey pairwise comparison indicates a statistical difference for the median RNAi silencing memory between N2 and *drh-1(bab552)* in N2: *p* = 1.1x10^−3^; between MY10 and *drh-1(bab552)* in N2: *p* < 1×10^−4^.

One gene involved in the complex responsible for foreign dsRNA recognition and degradation is *drh-1*, a RIG-I like DExD/H box helicase with dsRNA binding activity (Duchaine et al., 2006; Batachari et al., 2024; Consalvo et al., 2024). Studying variation in the sensitivity to Orsay virus infection among wild *C. elegans* strains, we previously found that a 159 bp natural deletion allele (*niDf250*) in the RIG-I like domain of DRH-1 was causally associated with hypersensitivity to viral infection and was present at an intermediate frequency in the species (Ashe, Bélicard, Le Pen, Sarkies et al., 2013) (Fig. 3A).

To test whether this *drh-1* allele affected RNAi memory, we reproduced this natural deletion by genome editing of the N2 genetic background, yielding allele *drh-1(bab552)*, and crossed it to the *mex-5p::ce-GFP::tbb-2* transgene. The RNAi efficacy was tested in this line along with the N2 and MY10 backgrounds, the latter a natural carrier of the deletion. We first observed that initiating RNAi using *E. coli* iOP50 transformed with pFF1 (see Methods) increased RNAi efficiency as even MY10 was almost fully silenced at G0 (Fig. 3B, Fig. S4). We also noted that, when placed in an N2 background, the partial deletion of *drh-1* not only reduced *gfp* RNAi memory but made animals somewhat less responsive to RNAi initiation (Fig. 3B–C). The *drh-1* deletion allele is maintained at intermediate frequency in the *C. elegans* species, particularly in Eurasia and Africa and is thus a common allele potentially affecting RNAi memory (and marginally, efficiency) in natural populations.

### Structural polymorphisms at the *eri-6/7* locus likely affect RNAi memory

Besides exogenous double-stranded RNAs, different endogenous small RNA pathways exist in *C. elegans*. In both cases, the primary small RNAs are amplified in secondary 22G small RNAs (Billi et al., 2014). Because factors required for the amplification of exogenous and endogenous small RNAs are shared, the different small RNA pools compete amongst each other for amplification (Houri-Ze’evi et al., 2016; Shukla et al., 2021; Karin et al., 2023). For instance, the endogenous piRNA pathway modulates the duration of exogenous RNAi memory: the inactivation of the piRNA pathway results in a longer exogenous RNAi memory (Shukla et al., 2021). Inactivating mutations in the ERGO-1 26G-RNA pathway enhance RNAi (Eri phenotype) (Kennedy et al., 2004; Lee et al., 2006; Fischer, Butler et al., 2008) but a potential effect on RNAi memory had not been tested.

**Figure 4:**
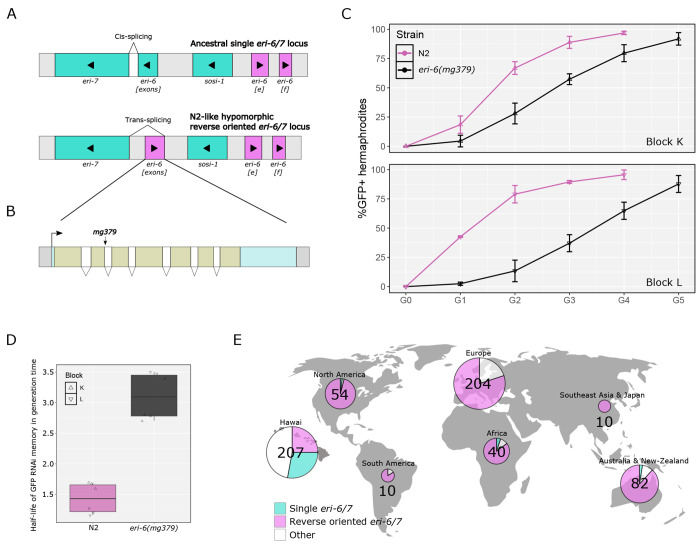
Competition between endogenous and exogenous small RNA pathways alters RNAi memory duration. **(A)** Diagrams depicting two commonly found gene structures of the *eri-6/7* locus in C. elegans wild strains (here additional introns within each large block are omitted). The ancestral state of this locus harbors a unique gene in the right to left orientation (colored in blue). The more recent N2-like structure of *eri-6/7* is thought to have arisen from a transposable element invasion, a resulting inversion of the first exons (pink orientation, left to right) forming the so-called *eri-6* part of the gene. This rearrangement of *eri-6* is predominant in isolates outside of Hawaii and is hypomorphic (Fischer, Butler et al., 2008; G. Zhang et al., 2024). **(B)** Diagram showing the detailed gene structure of *eri-6* as found in the N2 genetic background. The laboratory *mg379* allele is a splice-donor mutation. **(C)** RNAi memory assay (blocks K and L) of the *eri-6(mg379)* mutant, using the *mex-5p::ce-GFP* transgene. This *eri-6* reduction-of-function mutation decreases endogenous small RNA production, thus enabling a better amplification of 2° siRNAs corresponding to exogenous dsRNAs and leads to a longer RNAi memory. *E. coli* iOP50 was used to initiate *gfp* RNAi. **(D)** Boxplot showing estimated half-lives of GFP RNAi memory for N2 and *eri-6(mg379)* in blocks K and L. Glmm on these half-life values followed by a Tukey pairwise test indicates a statistical difference of median RNAi silencing memory between strains: *p* < 1x10^−4^. **(E)** World map presenting the percentage of isotypes possessing either one of the *eri-6/7* gene structures shown in (A) relative to the total number of isotypes by world region in CaeNDR. Other structures of this locus comprise, for example, a single *eri-6/7* gene without *sosi-1* and *eri-6[e][f]* genes, *eri-6[exons]* surrounded by remnants of the transposable element and an N2-like structure with duplicated *eri-6[exons* (G. Zhang et al., 2024). Created with BioRender.com.

The *eri-6/7* locus produces a helicase involved in the endogenous ERGO-1 26G-RNA pathway repressing retrotransposons and novel genes (Fischer et al., 2011; Fischer and Ruvkun, 2020). The gene has been shown to greatly vary in structure among *C. elegans* wild isolates (Fig. 4C), with an inversion of the *eri-6* part of the gene and further indels, impacting the splicing efficiency of the inverted *eri-6* exons to the remaining *eri-7* exons and the expression of their downstream targets (Fischer, Butler et al., 2008; G. Zhang et al., 2024).

We tested whether the laboratory-induced *eri-6(mg379)* splice site mutation (Fig. 4B) affected the duration of exogenous RNAi silencing memory. We found that this reduction-of-function mutation increased RNAi memory (Fig. 4A), possibly through the competition between small RNA pools for amplification. The natural rearrangements impacting the *eri-6/7* locus activity thus likely affect RNAi memory in *C. elegans*.

### Environmental effects on RNAi memory of *C. elegans* wild isolates

**Figure 5:**
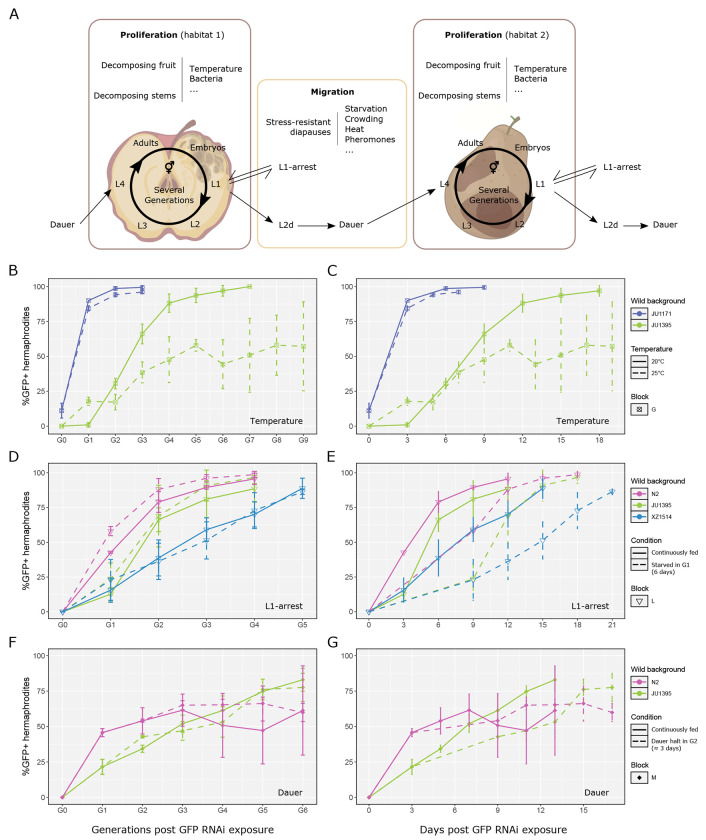
Impact of the environment on the duration of RNAi memory. **(A)** Schematic illustration of the “boom-and-bust” life cycle of *C. elegans* natural populations (created with BioRender.com). **(B-G)** Tests of temperature [B-C], L1 arrest [D-E] and dauer diapause [F-G] on RNAi memory duration using the *mex-5p::GFP* transgene, initiating RNAi against *gfp* with *E. coli* iOP50 bacteria transformed with pFF1. The results are plotted along the number of generations in [B, D, F] and number of days in [C, E, G]. For each block and condition, 3 replicates were run per strain. Graph lines represent means and bars the standard deviation. **(B-C)** Effect of temperature on RNAi memory in the JU1171 and JU1395 backgrounds (block G). Culturing JU1395 at 25°C increases duration of its RNAi memory in number of generations (glmm on half-lives of GFP RNAi memory in blocks C and G, *p* < 1x10^−4^) and in days (*p* = 5.3x10^−3^). No significant difference was observed for JU1171. See Fig. S2B for an additional experiment using the N2 and JU1395 backgrounds. **(D-E)** Effect of L1 arrest for 6 days at 20°C at the first generation after parental RNAi exposure for strains in the N2, JU1395 and XZ1514 backgrounds (block L). Analyzing the half-lives of the GFP RNAi memory of N2 and JU1395 in blocks K and L in a glmm framework, a significant reduction between control and starvation conditions were found in generations, *p* = 2,8x10^−3^ and 5x10^−4^, for N2 and JU1395, respectively, as well as a significant extension of RNAi memory in absolute time, *p* < 1x10^−4^ for each strain. **(F-G)** Effect of a ~3-day long dauer diapause at the second generation after GFP RNAi exposure (block M). No effect was found on GFP de-silencing dynamics when expressing the results in number of generations (glmm on half-lives, followed by Tukey’s test, *p* > 0.45 between fed and dauer condition for both N2 and JU1395). However, the RNAi memory was extended in number of days after passage through dauer in G2 in the JU1395 background (*p* = 5.6x10^−3^ between fed and dauer conditions). This experiment was performed at 25°C.

Having demonstrated a genetic component in the duration of GFP silencing, we investigated whether the environment may impact this duration. In natural contexts, *C. elegans* follows a boom-and-bust life cycle (Fig. 5A) and is subject to multiple biotic and abiotic constraints (Félix and Duveau, 2012; Frézal and Félix, 2015; Schulenburg and Félix, 2017). We focused on some of these relevant factors to assess their potential impact on RNAi memory: temperature, passage through diapause states (Fig. 5B–G), and bacterial environment (Fig. S2A). In order to keep RNAi efficiency constant across environmental conditions, we initiated GFP silencing memory as above until G0 and modified the conditions in which the subsequent generations were cultured.

We first compared RNAi memory at two temperatures, 20°C (temperature of previous assays) and 25°C, on two strains displaying different memory durations. We found that the JU1395 background strain transmitted GFP silencing for a higher number of generations at 25°C than at 20°C, whereas the higher temperature did not rescue the lack of memory of the JU1171 strain (Fig. 5B–C). Development occurs faster at 25°C compared to 20°C, therefore we tested whether the longer memory in number of generations corresponded to a similar memory in absolute time. Instead, the JU1395 background still displayed a longer memory at high temperature in absolute time (Fig. 5B–C). We further tested the RNAi memory of the N2 strain at 25°C versus 20°C. Similar to the JU1395 background, culturing N2 at the higher temperature resulted in an enhanced *gfp* RNAi memory, in number of generations and in absolute time (Fig. S2B).

When facing harsh conditions during post-embryonic development, *C. elegans* can arrest developmentally, and survive for several weeks without eating or reproducing (Hu, 2007; Baugh, 2013); when encountering new favorable conditions, normal growth can be restored (Fig. 5A). For example, the L1 larval arrest arises after hatching in the absence of food (Baugh, 2013). To reproduce this L1 developmental arrest, we let the first generation after GFP RNAi exposure hatch in NGM plates without *E. coli* and remain for six days at 20°C, as in Houri-Zeevi et al. (2021). L1 larvae were then fed again in standard conditions and RNAi memory assayed. This early L1 arrest slightly reduces RNAi memory for the three tested strains when counting in number of generations (Fig. 5D). Due to the time spent in L1 arrest, when plotting in absolute time, RNAi memory was increased (Fig. 5E).

The stress-resistant dauer diapause, commonly found in *C. elegans* natural populations (Schulenburg and Félix, 2017), corresponds to a specialized form of the early third larval stage (Hu, 2007). Experimentally, dauer larvae can be obtained in conditions of crowding, paucity of food and elevated temperature on young larvae (Karp, 2018). In order to keep the conditions of RNAi initiation constant, we induced dauer larvae by letting the first generation (G1) animals lay embryos without transfer to a new food source and selected for dauers after a few days (see Methods). Passage through dauer for 5 days in the second generation after RNAi initiation did not alter RNAi memory kinetics of the N2 and JU1395 strains (Fig. 5F). Again, taking into account the diapause delay, passage through dauer increased RNAi memory in absolute time (Fig. 5G). We further tested the long memory background XZ1514 with dauer induction at 20°C, reproducing this procedure at every generation after G2, and similarly did not see an effect of passage through dauer (Fig. S2C). Thus, we conclude that the dauer stage, like the early L1 arrest, does not strongly affect memory in number of generations and is able to maintain the memory of RNAi through the days of diapause.

Bacteria that are found naturally associated with *C. elegans* encompass many different genera (Dirksen et al., 2016; Samuel et al., 2016). We tested four such bacterial wild isolates, *Leucobacter* CBX151, *Chryseobacterium* JUb044, *Acinetobacter* BIGb0102 and *Comamonas* BIGb0172, selected for their ability to sustain *C. elegans* growth for multiple generations while being potential pathogens (Hodgkin et al., 2013; González and Félix, 2024). We found that RNAi memory of the JU1395 and JU1171 wild backgrounds was conserved relative to *E. coli* OP50, when cultured with these naturally-associated bacteria. An exception is the JU1395 strain, which when tested on *Leucobacter* CBX151 displayed a slight reduction in memory duration (Fig. S2A).

## Discussion

We here provided evidence for microevolutionary variation in small RNA inheritance in *C. elegans*. The duration of epigenetic inheritance can be treated as a quantitative trait in theoretical (Helanterä and Uller, 2010; Day and Bonduriansky, 2011) and experimental models of selection.

### Natural genetic variation in RNAi inheritance

Like RNAi efficiency itself (Pollard and Rockman, 2013; Chou et al., 2024), the small RNA inheritance trait is likely to have a polygenic basis. We provide evidence for polymorphisms in several genes being involved, potentially acting at distinct points in the inheritance mechanism. These may correspond to different parameters in mechanistic models of small RNA memory, such as that developed by (Karin et al., 2023), which models feedback amplification between small RNA and histone modifications, a resulting variation in mRNA level, and a queuing system of small RNAs. Different molecular mechanisms for the extinction of memory may be at stake as well (Shukla et al., 2021; Knudsen-Palmer et al., 2024). Furthermore, after RNAi initiation, two phases have been experimentally distinguished in transgenerational silencing, based on the mutation effect of different genes in the N2 background (Woodhouse et al., 2018): establishment of RNAi inheritance at the first generation and maintenance in further generations (Fig. S3).

The *drh-1* deletion allele seems to mildly diminish sensitivity to germline RNAi and severely prevents RNAi inheritance (Fig. 3B, Fig. S4A). Memory may be simply affected by the slightly lesser efficiency of the initial trigger, resulting in a less efficient initial amplification. Alternatively, competition between small RNA pools along the generations may be at stake, as seen with piRNAs or endosiRNAs, or as modeled by (Karin et al., 2023). DRH-1 is a cytoplasmic protein (Batachari et al., 2024), expressed in both somatic and germline cells (Wu et al., 2012). It recognizes preferentially blunt dsRNA (Consalvo et al., 2024) and is involved in viral RNA recognition as well as a small RNA response to mitochondrial stress (Mao et al., 2020). In an evolutionary context, *drh-1* polymorphism may be driven by these different phenotypes alongside its effect on RNAi memory.

The *set-24* gene codes for a SET and SPK-domain protein, recently characterized by Zeng, Furlan, Almeida et al. (2025). The SET-24 protein is localized to the germline nuclei. Through its interaction with the epigenetic regulator HCF-1 and chromatin remodeling complexes, SET-24 regulates H3K4me3 levels of hundreds of genes. Its dysregulation perturbates the pools of inherited small RNAs, which leads to impaired inheritance and a shorter RNAi memory (Zeng, Furlan, Almeida et al., 2025).

The *eri-6/7* locus quantitatively affects the maintenance of the memory, likely by competition between pools of small RNAs, as modeled by Karin et al. (2023), whereby amplification of endo-siRNAs requiring ERI-6/7 competes with other small RNA pools. The partial inversion of the *eri-6/7* locus n the N2 background results in a hypomorphic allele (G. Zhang et al., 2024), thus likely increasing RNAi memory. We note however that the only strain with an ancestral single orientation *eri-7* locus in our set, XZ1514 (G. Zhang et al., 2024), has a long memory so other variants elsewhere in the genome of this strain likely affect the trait. It is likely that the variation in duration of small RNA memory is highly polygenic in *C. elegans* wild strains.

### Environmental effects on RNAi inheritance

Given the boom-and-bust lifecycle of *C. elegans* with a dispersal between food patches in which the environment may differ (Félix and Duveau, 2012), we expected that starvation and passage through the dispersal dauer stage may erase the small RNA memory. However, of the different culture environments we tested during the memory phase, including dauer, only temperature variation resulted in a large change, with an enhanced RNAi memory at 25°C compared to 20°C. This effect of temperature appeared after one generation of initiation of the memory.

Houri-Zeevi et al. (2021) instead suggested that small RNA memory was ‘reset’ by different stressing environments. They found a shortened memory, from the first generation, after a transient heat shock (37°C). In contrast, they had previously shown that inheritance of silencing relied on the activity of the heat-shock transcription factor HSF-1 (Houri-Zeevi et al., 2020), an effect that could be relevant in our experiments. *hsf-1* overexpression leads to suppression of endogenous small RNA (sRNA) pathway components and, likely due to competition among sRNA pools, a higher proportion of silenced progeny. Using a 25°C temperature as a trigger rather than an environment during the memory phase, others studies showed that this temperature altered siRNA pools for 3-4 generations (Schott et al., 2014; Belicard et al., 2018) and de-repressed a silenced multi-copy fluorescent reporter for over 10 generations (Klosin et al., 2017). Temperature is thus an ecologically relevant factor initiating and modulating small RNA memory.

Concerning L1 stage starvation, Houri-Zeevi et al. (2021) reported a ‘resetting’ of silencing memory, analyzing statistically each successive generation as if independent. Following the same experimental procedure, and analyzing instead half-lives of silencing, we observed a statistically significant yet mild difference on RNAi memory of *C. elegans* between fed and starved conditions. Moreover, after halting development in the L1 or dauer stages, silencing is maintained for a longer time in number of days. Knowing that *C. elegans* in the wild remains in the dauer stage possibly for weeks in between nutrient-rich sources, questions arise regarding the ecological relevance of memorizing conditions of a previous environment.

## Conclusions and perspectives

A transient epigenetic memory may be advantageous in environments that change on the same timescale, with correlation between successive environments (Jablonka et al., 1995; Lachmann and Jablonka, 1996; Rivoire and Leibler, 2014). In that case, the frequency of environmental change and the life cycle duration of the organism will determine whether multigenerational memory is advantageous. One can imagine the evolution of different memory durations, with cases relying more on plasticity within a generation than on inheritance. Using *C. elegans* experimental evolution, (Dey et al., 2016) showed that parental effects could be selected in sequences of environments that vary at every generation. Our findings of polymorphisms that affect small RNA inheritance will allow to test which environmental sequences may favor one or the other allele. In addition, polymorphisms affecting RNAi memory duration may be selected for a pleiotropic effect on another trait. The prevalence of different forms of non-genetic inheritance across species highlights the importance of evaluating experimentally how it itself evolves and might impact the course of evolution.

## Materials and methods

### *C. elegans* culture and strains

*C. delegans* was cultured on 55 mm NGM agar plates (Stiernagle, 2006) seeded with 100 μl of a saturated culture of *E. coli* OP50 grown overnight in Luria Broth (LB) at 37°C, except if otherwise indicated. *C. elegans* wild isolates were obtained from the Caenorhabditis Genetics Center (CGC), Michael Ailion, Erik Andersen, Leonid Kruglyak or our own collection. The *set-24(bab562)* and *drh-1(bab552)* mutants were engineered using CRISPR/Cas9 editing by the CNRS SEGICel facility (Lyon, France), mimicking the natural alleles characterized in Frézal et al. (2018) and Ashe, Bélicard, Le Pen, Sarkies et al. (2013), respectively. The *eri-6(mg379)* mutant (Fischer et al., 2008) was obtained from the CGC. Bleaching of cultures was performed as in Hibshman, Webster et al. (2021).

### *E. coli* strains and plasmids

The *gfp* targeting sequence directed against the *pie-1p::GFP* transgene was obtained from Eric Miska’s laboratory and inserted into the L4440 vector. The *gfp* targeting sequence directed against the *mex-5p::ce-GFP* transgene was inserted into plasmid L4440 (Timmons et al., 2001), yielding plasmid pMNK25. The NotI-KpnI digested insert was subcloned into the T444T plasmid (Sturm, Saskoi et al., 2018), yielding plasmid pFF1, the latter harboring transcriptional terminators (Table S4). The RNAi clone that was used is indicated for each experiment. In our first experiments, *E. coli* bacterial strains HT115 was used transformed with plasmid pMNK25 and in later experiments, *E. coli* iOP50 was used with pFF1. The latter combination seems to result in a stronger RNAi efficiency, but the overall variation among strains is comparable for all clones.

### Bacterial strains

The non-*E. coli* bacteria were originally isolated from different *Caenorhabditis* collected in the wild. CBX151 *Leucobacter* was found infecting a *Caenorhabditis* collected in Cape Verde (Hodgkin et al., 2013). JUbO44 *Chryseobacterium* was isolated from Santeuil, France; BIGb102 *Acinetobacter* and BIGbl72 *Comamonas* were collected in decomposing apples in Orsay, France (Samuel et al., 2016). These bacteria were grown in LB liquid culture for 1 16 or 1 32 h with 220 rpm agitation at 22°C for CBX151 or 28°C for the other bacteria.

### Introgression of the *pie-1p::GFP::H2B::pie-1* transgene

We initially used the *mjls31[pie-1p::GFP::H2B]* transgene (Ashe, Sapetschnig et al., 2012), introduced by MosSCI (Frøkjær-Jensen et al., 2008) in the reference strain N2 on chromosome II at 8.4 Mb, yielding strain SX461 (Ashe, Sapetschnig et al., 2012). As this transgene is easily silenced, we first desilenced its expression using *mut-7* RNA interference. We then introgressed the transgene in several wild isolates by at least six rounds of backcrossing to the target wild genetic background, using SX461 males in the first cross. The primers used to verify the genotype of the different chromosomes are in Table S5 for JU1171, MY10 and JU775, with specific attention to the presence of *set-24(mfP23)II* in the MY10 genetic background; genotyping was not performed for JU1395 because of its close relatedness to N2. As this transgene was introduced in different wild isolates through backcrosses (Ashe, Sapetschnig et al., 2012), a N2 genomic segment linked to it is also present and of a different length for each backcross. As these N2 genomic regions could in principle affect RNAi memory compared to the target wild genomic background, we also used CRISPR/Cas-9 genome editing to introduce the transgene at a given locus, circumventing this problem.

### Introduction of the *mex-5p::ce-GFP::tbb-2 3’UTR* transgene by genome editing

A CRISPR-Cas9 protospacer was selected (TCCGTGTCTTACTACTGTA) in a conserved region on chromosome *I* that is permissive for germline expression (El Mouridi et al., 2022). The protospacer was added to an sgRNA(F+E) scaffold (Chen et al., 2013) and ordered as a gene fragment (Twist Bioscience, CA, USA). The gene fragment was subsequently cloned into an empty ampicillin resistant vector using Gibson assembly (Gibson et al., 2009) to give pMDJ344. An expression vector containing a *mex-5* promoter (pCFJ645) (Zeiser et al., 2011), a nuclear *C. elegans*-optimized GFP (pCFJ2389) (Aljohani et al., 2020), a *tbb-2* 3’UTR (pCM1.36) (Merritt et al., 2008), and a hygromycin resistance cassette (pCFJ767) (Radman et al., 2013) were cloned using a multi-site Gateway reaction (Invitrogen cat. no. 12538200) to give pMDJ343. Roughly 250 bp homology arms from either end of the selected CRISPR-Cas9 cut site were added to pMDJ343 using Gibson assembly (Gibson et al., 2009) to make the final repair template pMDJ346. The resulting lines are listed in Table S1.

A similar procedure was performed exclusively on an N2 genetic background, this time using a (CCGTGGAATCAAGTTAATC) CRISPR-Cas9 protospacer in a conserved region of chromosome V. By using the same methodology as stated above, the resulting gene fragment cloned into an empty ampicillin resistant vector gave pMDJ345. The adding of 250 bp homology arms from either end of this chromosome V cut site to the *mex-5p::ce-GFP::tbb-2* transgene and its hygromycin resistant cassette gave pMDJ347. This insertion was used to cross the *eri-6* mutation located on chromosome I.

Injection mix contained a codon-optimized Cas9 expression vector (pCFJ2474) (Aljohani et al., 2020) at 25 ng/μl, the sgRNA vector (pMDJ344) at 5 ng/μl, the repair template (pMDJ346) at 10 ng/μl, a linearized histamine marker (pSEM238) (El Mouridi et al., 2021) at 10 ng/μl, a pan-muscular mCherry marker (pSEM235) (El Mouridi et al., 2020) at 10 ng/μl, and a DNA ladder (1 kb plus, Invitrogen) at 40 ng/μl to make up a final mix concentration of 100 ng/μl. 10-20 young adults were injected for each wild isolate as previously described (Mello et al., 1991) and placed at 20°C. 500 μl of hygromycin (4 mg/ml) was added three days post injection to select for extrachromosomal array animals (Radman et al., 2013). Once plates starved (7 days post injection), 500 μl of histamine (500 mM) were added to paralyze extrachromosomal array animals (El Mouridi et al., 2021). Single copy insertion lines are characterized by hygromycin resistance, insensitivity to histamine, lack of somatic mCherry fluorescence, and germline *ce-gfp* expression.

### Comparison between the transgenes

The *pie-1p::GFP* transgene possesses three introns and four exons. Sequence of *gfp* in the *mex-5::ce-GFP* transgene is around 1.75 kb and possesses six introns and five exons.

The *mex-5p::ce-GFP* transgene was custom-built for this work. It includes an engineered *gfp* sequence that was designed to lack piRNA homology sites and favor transgene expression (Aljohani et al., 2020). We cannot rule out that the piRNA loci differ among the *C. elegans* wild isolates and that for example some of the long memory strains possess piRNAs targeting this transgene. However, we note that the same ranking of wild strains regarding RNAi memory was observed with the *pie-1p::GFP* transgene, which has a quite different sequence, which only matches that of the *mex-5p::ce-GFP* transgene on 571/792 nucleotides along the mRNA. For RNAi, we used *E. coli* produced dsRNAs (Table S4) with a perfect match against the length of the corresponding mRNA.

### Culture plates for RNAi interference

Empty 55 mm Petri dishes were poured with autoclaved NGM medium supplemented with a 2 μm filtered IPTG solution to obtain a final concentration of 1 mM. Plates were left to dry overnight and covered at room temperature. In parallel, liquid cultures of dsRNA vector containing-bacteria were incubated for ~16 hrs at 37°C with agitation at 220 rpm. The next day, the bacterial liquid cultures were used to seed now solidified NGM-ITPG plates with 100 μL and left to dry overnight covered at room temperature. This process was repeated for the two generations of exposure to RNAi (G-1 and G0).

### GFP silencing memory assay

Stock populations of GFP+ nematodes were maintained for at least three generations at 15°C before starting an experiment. Three replicates were conducted in parallel for each. RNAi was initiated 20°C by randomly picking three L4 stage individuals from the stock populations onto RNAi plates. Three L4 stage larvae derived from these founders (designated generation G-1) were randomly chosen for transfer onto fresh RNAi plates. In block A, 60 L4 larvae of the next (G0) generation were transferred from the RNAi memory environment to standard conditions, without RNAi trigger. For all other blocks, the G0 adults were treated with a “bleach” solution (Hibshman et al., 2021) to break down their cuticle and isolate their embryos, thus killing dsRNA expressing bacteria. Embryos were then deposited onto standard 20°C NGM plates seeded with saturated OP50 *E. coli*, unless indicated otherwise. These embryos were designated as generation G1. At each generation, 60 random L4 stage individuals were passed to produce the next generation and the remaining population chunked onto a fresh new NGM-OP50 plate to avoid starvation. The next day, the chunked population was scored and the transferred individuals were removed and their progeny allowed to grow.

Changes in the environment were performed during the memory phase, thus after the initiation step of parental RNAi exposure at 20°C in G-1 and G0.

#### Temperature:

After the initiation step, subsequent generations were cultured at 25°C or 20°C on standard NGM medium seeded with 100 μL of saturated *E. coli* OP50 culture.

#### Bacteria:

After the initiation step, subsequent generations were cultured at 20°C in standard NGM medium seeded with 100 μL of saturated cultures of the tested bacteria.

#### Starvation:

After the initiation step, the bleach-treated embryos were deposited onto an empty NGM plate and left to starve for 6 days at 20°C. At this time point, L1 larvae were collected in M9 solution, centrifuged 1 min at 3000 rpm and transferred to standard culture plates to resume their development and continue the GFP silencing memory experiment as described above.

#### Dauer:

In order to keep the conditions of RNAi initiation constant, we induced dauer larvae by letting the first-generation animals lay embryos without transfer to a new food source. Specifically, after initially transferring 60 L4 stage G1 individuals onto new plates for the fed control, the remaining population was separated in two, half being fed for scoring of the G1 generation and the other half left on the plate. The latter was then placed at either 20°C or 25°C for 7 and 5 days, respectively, in order to obtain a mix of L1/L2 arrest and dauer larvae of the next generation. 1% sodium dodecyl sulfate (SDS) was then used to filter out dauer individuals from the rest by ~20 min incubation with mellow agitation (Karp, 2018). Surviving dauer larvae were then deposited back onto standard culture plates to resume development and continue the assay.

### Microscopy and GFP expression quantification

Animals harboring the *pie-1p::GFP* transgene were scored using a Zeiss AxioImager M1 microscope with a x63 objective and a Semrock GFP-1828A-000 filter. Animals harboring the *mex-5p::ce-GFP* transgene were scored using a Nikon AZ100 fluorescence microscope with a 5x objective and level 3 zoom and a Semrock GFP-3035D filter. Hermaphrodite adults were collected using M9 solution and pelleted waiting ~2 minutes. A 15 μL drop of pelleted nematodes was deposited on a microscope slide containing a thin layer of 4% agar noble gel supplemented with 100 μM of NaN_3_. Around 100 individuals were scored for each replicate using no transillumination light. During the silencing recovery period, with the *pie-1p::GFP* transgene, nematodes were considered “OFF” when both arms were silenced or “ON” when at least one mature oocyte presented nuclear fluorescence. With the *mex-5p::ce-GFP* transgene, intensity and site of GFP expression varied among individuals leading us to sort nematodes according to their GFP pattern and brightness levels into “OFF”, “DIM” or “ON” categories. OFF individuals were those showing no nuclear fluorescence in their gonad, DIM individuals were those with varied patterns of expression but no nuclear fluorescence in their proximal gonad and ON individuals expressing GFP+ meiotic oocyte nuclei. Results with these three categories are shown in Table S2 and Figure Sn. The OFF and DIM categories were grouped for plotting and analysis as we considered that DIM individuals were still inheriting RNAi.

### Half-life of GFP RNAi memory and statistical analysis

We calculated the GFP RNAi memory half-life values arithmetically based on the raw scoring counts presented in Table S2. For every replicate of very strain in every block, we selected the two scoring time points for which the percentage of GFP-positive individuals were the closest below and above 50, respectively, and calculated the corresponding line equation. Based on this equation, we obtained the theoretical median value of GFP RNAi memory allowing us to generate the boxplots shown in Fig. 1E, Fig. 2B, Fig. 3C, Fig. 4D and Fig. S1 and to conduct statistical analysis. For values oscillating around the median line, a smoothing was realized picking scoring time points further apart. Raw values and indications can be found in Table S3.

Generalized linear mixed model (GLMM) were fit using the glmmTMB (v.1.1.10) package in the R version 4.2.2 (2022-10-31) to compare differences in half-life of GFP RNAi memory across strains or environments.

The model we used to test for the effect of genetic variation was:

model_1<−glmmTMB(Value~Strain+(1|Block)+(1|Block:Replicate),family=Gamma(link="log"),data=data)

The model we used to test for the effect of an environmental variable was:

model_2<-glmmTMB(Value~Strain*Environment+(1|Block)+(1|Block:Replicate),data=data)

We used the *emmeans* package (v.1.10.6) and Tukey’s post-hoc test for pairwise comparison, either among strains:

emmeans(model,pairwise~Strain,adjust="tukey")

or between environments for a given strain:

emmeans(model,pairwise~Environment|Strain,adjust="tukey")

For blocks realized only once, the random variable (1| Block) was removed from the model. All models were validated by fitting the DHARMa package (v.0.4.7) simulations.

### Haplotype tree building and genetic relatedness

We downloaded the 611 hard-filtered Variant Call Format (VCF) file from CaeNDR (20220216 release) (Cook et al., 2017) from which we extracted the data for our eight strains of interest. We converted this VCF file to a PHILIP file using the *vcf2phylip.py* script (Ortiz, 2019). The haplotype network was generated using this PHILIP file and the SplitsTree software (v6.4.13) (Huson and Bryant, 2006).

Presence of the *drh-1* 159 bp deletion among the 611 hard-filtered isotypes was detected using BCFtools (v1.9) and manually checked via CaeNDR’s genome browser tool. Sampling location for each *C. elegans* isotype was downloaded from CaeNDR (20231213 data release).

## Supplementary Material

1
