# Supplementary material for: Intraspecific variation in the duration of epigenetic inheritance
Source: bioRxiv. 2025 Jun 7:2025.06.04.657799. Preprint. [Version 1] doi: 10.1101/2025.06.04.657799 (PMC12258893; doi:10.1101/2025.06.04.657799)
Supplement: 1 [file NIHPP2025.06.04.657799v1-supplement-1.pdf]

bioRxiv preprint doi: https://doi.org/10.1101/2025.06.04.657799; this version posted June 7, 2025. The copyright holder for this preprint (which was not certified by peer review) is the author/funder, who has granted bioRxiv a license to display the preprint in perpetuity. It is made available under aCC-BY 4.0 International license.

Supplementary figure legends

Figure S1: The rank order of memory duration of wild background strains is overall conserved when using the two distinct GFP transgenes.
(A) GFP silencing memory assay comparing in parallel the RNAi memory duration of the reference strain N2 with either the *pie-1p::GFP::H2B* or the *mex-5p::ce-GFP* transgenes. *E. coli* HT115 with the relevant *gfp* sequence clone for each transgene was fed to initiate RNAi against *gfp*. Three biological replicates were run for each strain, scoring the proportion of GFP-positive (GFP+) animals at each generation. On the graph, the lines follow the means of three replicates and error bars represent their standard deviation (SD).
(B) Boxplot showing the GFP RNAi memory half-lives of a N2 genetic background with the *mex-5p::ce-GFP* or *pie-1p::GFP* transgenes. Half-lives were estimated from the scoring data shown in (A).
(C) Diagram depicting the two *gfp* transgenes used in this study. The *pie-1* promoter is 2 kb long and not represented to scale. The two transgenes strongly differ in terms of cis-regulatory and coding sequences.
(D) Two independent experiments assaying the RNAi memory duration of the same wild isolates containing either the *pie-1p::GFP::H2B* (Block A) or *mex-5p::ce-GFP* (Block C, also plotted in Fig. 2) transgenes. For both blocks, *E. coli* HT115 was fed to initiate RNAi against *gfp*. As the *pie-1p::GFP* experiment only tested the MY10, JU1171, JU775, N2 and JU1395 backgrounds, only the relevant strains are plotted for *mex-5p::ce-GFP*. The variation in memory of the wild strains is overall conserved when using the two distinct GFP transgenes.
(E) Boxplots showing the GFP RNAi memory half-lives of the MY10, JU1171, JU775, N2 and JU1395 backgrounds with either GFP transgene. As expected, the memory is longer with the *pie-1p::GFP::H2B* where piRNA recognition sites have not been avoided.

Figure S2: Effect of additional culture environments on the duration of RNAi memory.
(A) Three different experiments (blocks H, I and K) testing for the effect of *Chryseobacterium* JUb044, *Acinetobacter* BIGb102, *Comamonas* BIGb172 and *Leucobacter* CBX151 on RNAi memory. Here the moderate-memory strain JU1395 or low-memory strain JU1171 containing the *mex-5::ce-GFP::tbb-2* transgene were fed with *E. coli* iOP50 to initiate *gfp* RNAi. No striking differences were observed in RNAi memory profile between *E. coli* OP50 or naturally associated bacteria except for a reducing effect of CBX151 on the nematode strain JU1395. Statistics comparing estimated GFP memory half-lives of a given strain in different bacterial condition to the OP50 control: glmm followed by Tukey's comparison against OP50: $p>0.06$ for each bacterial strain on JU1171 and $p>0.3$ for each bacterial strain on JU1395 except with CBX151, $p = 0.02$. As no major delay was seen in development or egg laying under these conditions, generation time and absolute time are equivalent.
(B) The panels correspond to the same block L (distinct from the experiment shown in Figure 4B-C) testing for effect of temperature on RNAi memory in the strains JU1395 and N2 containing the *mex-5p::ce-GFP::tbb-2* transgene and fed with *E. coli* iOP50 to initiate *gfp* RNAi. Data were plotted either in number of generations or days after RNAi initiation.
(C) Independent block J testing for the effect on RNAi memory kinetics of dauer diapause induced at different generations. The long-memory strain XZ1514 containing the *mex-5p::ce-GFP::tbb-2* transgene was fed with *E. coli* iOP50 to initiate *gfp* RNAi. This experiment was performed at 20°C. Dauer individuals of a given generation were induced by letting parents of the previous generation lay eggs and not re-supplying plates with *E. coli* OP50. Individuals

bioRxiv preprint doi: https://doi.org/10.1101/2025.06.04.657799; this version posted June 7, 2025. The copyright holder for this preprint (which was not certified by peer review) is the author/funder, who has granted bioRxiv a license to display the preprint in perpetuity. It is made available under aCC-BY 4.0 International license.

were left for ~3 days in the dauer stage. This was performed at every generation by separating in half the control individuals as population to be scored (fed) or population to lay (not fed) (see Methods). Dauer larvae were selected by incubation of the whole population in 1% sodium dodecyl sulfate (SDS) for ~20 minutes at room temperature with moderate agitation before been deposited back onto standard NGM-OP50 plates to resume development. The dauer diapause does not greatly affect further GFP silencing memory dynamics when expressed in number of generations.

Figure S3: Sensitivity to RNAi exposure is lower in the *C. elegans* MY10 strain but the *set-24* mutation in the N2 background only affects memory.
Block F including *gfp* silencing dynamics during RNAi initiation. Starting from a 15°C stock population where every individual expresses GFP fluorescence, the two-generation (G-1 and G0) of GFP RNAi exposure leads to full GFP silencing except in the MY10 strain. Are shown in the graph the different phases of the *gfp* silencing inheritance process with initiation of *gfp* RNAi, establishment of RNAi inheritance, and maintenance of RNAi inheritance.

Figure S4: Efficiency of *gfp* RNAi initiation in wild strains and the *drh-1* mutant.
(A) Block N showing the dynamics of *gfp* silencing (generations G-1 and G0) in the MY10, JU1171, N2, *drh-1(bab552)* in N2, and JU1395 backgrounds harboring the *mex-5p::ce-GFP* transgene and fed with *E. coli* iOP50 for RNAi initiation. The *drh-1* natural deletion allele reduces both responsiveness to *gfp* RNAi as well as RNAi memory.
(B) Microscopy images illustrating GFP silencing and desilencing dynamics of wild isolates through time. GFP microscopy images from block N taken in parallel with the scoring. Before *gfp* RNAi, stock populations of all four natural strains show identical patterns of GFP expression. Use of iOP50 with a T444T cassette seems to enhance efficiency of RNAi as even MY10 is almost fully silenced for *gfp* at G0. At the first generation after RNAi exposure, almost the whole populations of MY10 and JU1171 recover fluorescence while populations of N2 and JU1395 retain some silencing. Dots in the pharynx of silenced individuals are due to autofluorescence.

bioRxiv preprint doi: https://doi.org/10.1101/2025.06.04.657799; this version posted June 7, 2025. The copyright holder for this preprint (which was not certified by peer review) is the author/funder, who has granted bioRxiv a license to display the preprint in perpetuity. It is made available under aCC-BY 4.0 International license.

Supplementary tables

Table S1: List of nematode strains used in this paper.
List of wild *C. elegans* isolates and their derived transgene-modified counterparts with their origin and method of construction.

Table S2: Raw scoring data of GFP silencing memory assay.
Each block corresponds to an independent experiment. DIM and OFF individuals were pooled for simplicity in the graphs. Plasmid code names and bacteria used for GFP RNAi exposure are detailed in Table S4.

Table S3: Values of GFP RNAi memory half-lives.
Half-lives of GFP memory were calculated based on the raw scoring counts presented in Table S2 (see methods for procedure). A slash mark in the column for half-lives counted as days corresponds to cases with no difference between the time expressed in generation number or in days. For rare line plots values crossing the 50° value twice, a smoothing of the linear RNAi memory tendency was performed, and indicated by an arrow showing the selected data points.

Table S4: Bacterial strains and transgenes used in this paper.
The first sheet shows the list of *C. elegans* naturally associated-bacteria used as testing environments as well as artificial *E. coli* strains used for RNAi induction. The second sheet shows the sequences of *gfp* transgenes and their corresponding targeting dsRNA.

Table S5: Genotyping of the *pie-1p::GFP* transgene introgressions.
As the JU1395 and N2 genetic backgrounds are closely related to each other, we only used primers that could distinguish deletions in the MY10, JU775 and JU1171 isolates compared to N2.
